# *Escherichia coli* lipoprotein binds human plasminogen via an intramolecular domain

**DOI:** 10.3389/fmicb.2015.01095

**Published:** 2015-10-07

**Authors:** Tammy Gonzalez, Robert A. Gaultney, Angela M. Floden, Catherine A. Brissette

**Affiliations:** Brissette Laboratory, Department of Basic Sciences, University of North Dakota School of Medicine and Health Sciences, Grand ForksND, USA

**Keywords:** plasminogen, lipoprotein, *E. coli*, binding

## Abstract

*Escherichia coli* lipoprotein (Lpp) is a major cellular component that exists in two distinct states, bound-form and free-form. Bound-form Lpp is known to interact with the periplasmic bacterial cell wall, while free-form Lpp is localized to the bacterial cell surface. A function for surface-exposed Lpp has yet to be determined. We hypothesized that the presence of C-terminal lysinses in the surface-exposed region of Lpp would facilitate binding to the host zymogen plasminogen (Plg), a protease commandeered by a number of clinically important bacteria. Recombinant Lpp was synthesized and the binding of Lpp to Plg, the effect of various inhibitors on this binding, and the effects of various mutations of Lpp on Lpp–Plg interactions were examined. Additionally, the ability of Lpp-bound Plg to be converted to active plasmin was analyzed. We determined that Lpp binds Plg via an atypical domain located near the center of mature Lpp that may not be exposed on the surface of intact *E. coli* according to the current localization model. Finally, we found that Plg bound by Lpp can be converted to active plasmin. While the consequences of Lpp binding Plg are unclear, these results prompt further investigation of the ability of surface exposed Lpp to interact with host molecules such as extracellular matrix components and complement regulators, and the role of these interactions in infections caused by *E. coli* and other bacteria.

## Introduction

Murein lipoprotein (Lpp) —originally discovered in *Escherichia coli*—is the largest protein substituent, by molarity, in many species of Gram-negative bacteria (Braun and Sieglin, 1970; Braun and Wolff, 1970; Neidhardt and Curtiss, 1996; Sha et al., 2004; van Lier et al., 2014). The characterized function of Lpp is to anchor the outer membrane to the bacterial cell wall, aiding in stability and durability of the bacterial cell as a whole. Functional, bound-form Lpp is attached via its lipid moiety to the periplasmic leaflet of the outer membrane where the lipoprotein can be covalently attached to peptidoglycan (PG; Bernstein, 2011). However, a second population of Lpp exists in *E. coli*: that of a free-form (non-PG-bound) Lpp (Inouye et al., 1972). The subcellular localization of free-form Lpp has recently been described (Cowles et al., 2011). Current data suggest that free-form Lpp transverses the outer membrane such that the protein’s carboxyl terminus is exposed while the amino terminus remains unavailable—either remaining in the periplasm or becoming embedded within the outer membrane itself (Bernstein, 2011; Cowles et al., 2011).

A function for this free-form Lpp has yet to be determined. It has been posited that this population may serve as storage for future use as bound-form Lpp, although this hypothesis remains untested (Cowles et al., 2011). Our laboratory has an interest in bacterial lipoproteins as many have been characterized with important roles in adhesion and immune evasion (El-Hage et al., 2001; Kovacs-Simon et al., 2011; Zückert, 2014). We noted a striking feature on the proposed model of Lpp membrane insertion: the presence of C-terminal lysine residues (Shu et al., 2000). C-terminal lysines on bacterial lipoproteins have been shown to be important in the binding of the specific host protease precursor plasminogen (Plg), and many characterized Plg-binding proteins have this motif (Miles et al., 1991; Fulde et al., 2013). Plg is a serine protease present in serum as an inactive proenzyme. Plg is converted by tissue-type plasminogen activator (tPA) or urokinase plasminogen activator (uPA) to active plasmin (Fulde et al., 2013). Plasmin’s normal function is to aid wound repair and clot degradation; as it has a critical role in host fibrinolysis and extracellular matrix remodeling, plasmin’s activity is tightly controlled. Binding of Plg by mammalian Plg receptors is mediated by lysine-binding Kringle domains (Wiman et al., 1979). Binding of Plg to a mammalian receptor, fibrin clot, or a bacterial cell facilitates its activation to plasmin and makes the molecule less susceptible to inactivation (Lähteenmäki et al., 2001). Although plasmin’s normal function is to aid wound repair and clot degradation, many bacterial pathogens are able to hijack the host Plg/plasmin system and use its protease activity for dissemination from the original site of infection or to evade host immune response (Suomalainen et al., 2007; Bhattacharya et al., 2012; Önder et al., 2012). Indeed, *E. coli* possesses Plg-binding proteins (Parkkinen and Korhonen, 1989; Sjöbring et al., 1994).

In this study, we sought to determine the potential for Lpp to act as a Plg-binding protein, and the mechanisms on which this interaction depends, originating with the hypothesis that the C-terminal lysines facilitate this interaction. In summary, we found that Lpp was able to bind this host protein, albeit via an unexpected mechanism. In doing so, we also raise questions regarding the hypothesized orientation of free-form Lpp that we hope will contribute to future discussions regarding this abundant and interesting lipoprotein.

## Materials and Methods

### Alignment

The alignment of *E. coli* Lpp was performed via clustalW2 (Larkin et al., 2007) with the following accession sequence numbers: *E. coli*- WP_000648420.1; *Salmonella enterica*- AEF07247.1; *Shigella flexneri*- ABF03869.1; *Klebsiella pneumoniae*- EYB76101.1. For the rLPP and PDHB peptide alignment, sequences used were those presented in the paper. All parameters used were program defaults. Residues are annotated as follows: : = conservation of strong groups, • = conservation of weak groups, and **^∗^** = fully conserved residue.

### rLpp and Mutant Protein Production

Lipoprotein lacking the N-terminal 21 residues was cloned into the pET200 expression vector (Life Technologies; Carlsbad, CA, USA). Resultant colonies were screened by PCR, and correct insertion into the vector plasmid was confirmed via bi-directional Sanger sequencing (Davis Sequencing). To produce proteins, plasmids with the appropriate inserts were transformed into the BL21-Star strain *E. coli* (Life Technologies), and single colonies were transferred to an overnight culture of super broth (SB). The following day, inoculated SB was diluted 1:100 in fresh SB, and cells were grown to an optical density of approximately 0.5, after which time the cells were stimulated with 0.3 mM IPTG for 3–4 h. Cells were spun down, re-suspended in binding buffer (100 mM HEPES, 10 mM imidazole, 1 mg/mL lysozyme pH 7.5) for 1 h. Cells were subsequently lysed with a sonic dismembrator Model 705 (Fisher Scientific; Waltham, MA, USA) with the following protocol: 15 s at amplitude 100, 30 s rest for a total of eight cycles in an ice-water bath. Lysates were centrifuged and the soluble fraction was transferred to a new tube with 1 volume Magne-His beads (Promega; Madison, WI, USA) per 20 volumes of lysate. Lysates were allowed to interact with the beads for at least 30 min, after which time the supernatants were removed while beads were sequestered via magnetic stands and new binding buffer was added for 30 min to wash cells. This step was repeated twice for a total of three washes. To recover proteins, elution buffer (1 M imidazole, 100 mM HEPES, pH 7.5) was added to the beads and also allowed to act for at least 30 min. After removal of this buffer, this elution step was repeated once. Proteins in the elution were dialyzed into PBS with 3 kDa molecular weight cutoff dialysis cassettes (Life Technologies).

To produce mutants of Lpp, two techniques were employed, both of which use the primers detailed in **Table 1**. For C-terminal truncations and individual nucleotide substitutions, site-directed mutagenesis (SDM; Agilent; Santa Clara, CA, USA) was used to generate a premature stop codon in desired locations. Primers for this protocol were designed at the manufacturer’s website^1^, and the protocol followed was as described by the manufacturer. Briefly, parent plasmid was amplified with the SDM primers, the reaction was treated with DpnI provided in the kit, and the DNA was transformed into XL1 Blue *E. coli*, as recommended. Bacteria were plated on LB agar supplemented with kanamycin, and resultant colonies were moved into overnight culture. Plasmids for the culture were purified and sent for bi-directional Sanger sequencing as above to verify mutation of the desired nucleotides.

**Table 1:** A list of primers generated in this study and their functions.

Primer name	Sequence (5′–3′)	Function/peptide generated (also, see **Table 2**)
lpp pET For	CAC CTC CAG CAA CGC TAA AAT CG	Clone recombinant lipoprotein (lpp) into pET200 expression vector
lpp pET Rev	TTA CTT GCG GTA TTT AGT AGC	
lppΔ10CF	GCT CGT GCT AAC CAG CGT TAG GAC AAC ATG GCT ACT	Insert premature stop codon to remove 10 residues from Lpp C-term
lppΔ10CR	AGT AGC CAT GTT GTC CTA ACG CTG GTT AGC ACG ACG	
lppΔ30CF	CTG AGC AAC GAC GTG TAG GAC ATG CGT TCC GAC	Insert premature stop codon to remove 30 residues from Lpp C-term
lppΔ30CR	GTC GGA ACG CAT TGC CTA CAC GTC GTT GCT CAG	
lppΔ10NF	CCC TTC ACC TCT GAC GTT CAG ACT CTG AAC	Overlap deletion PCR to delete N-term 10 residues from Lpp
lppΔ10NR	AAC GTC AGA GGT GAA GGG ATG ATC CTT ATC	
lppΔ20NF	CCC TTC ACC GAC CAG CTG AGC AAC GAC GTG	Overlap deletion PCR to delete N-term 20 residues from Lpp
lppΔ20NR	CAG CTG GTC GGT GAA GGG ATG ATC CTT ATC	
lppΔ30NF	CCC TTC ACC CGT TCC GAC GTT CAG GCT GCT	Overlap deletion PCR to delete N-term 30 residues from Lpp
lppΔ30NR	GTC GGA ACG GGT GAA GGG ATG ATC CTT ATC	
lppK19 A For	GCT CAG CTG GTC AAC TGC AGC GTT CAG AGT CTG AAC G	Mutate lysine at site 19 in rLpp to alanine
lppK19 A Rev	CGT TCA GAC TCT GAA CGC TGC AGT TGA CCA GCT GAG C	
LppQ14A N17A For	CGA TCA GCT GTC TTC TGA CGT TGC GAC TCT GGC CGC TAA AGT TGA CCA G	Mutate both glutamine (site 14) and asparagine (site 17) to alanines
LppQ14A N17A Rev	CTG GTC AAC TTT AGC GGC CAG AGT CGC AAC GTC AGA AGA CAG CTG ATC G	
lppL16R A18R For	CTT CTG ACG TTC AGA CTC GGA ACC GTA AAG TTG ACC AGC TGA G	Mutate both leucine (site 16) and alanine (site 18) to arginines
lppL16R A18R Rev	CTC AGC TGG TCA ACT TTA CGG TTC CGA GTC TGA ACG TCA GAA G	
lppA18K For	GTT GCT CAG CTG GTC AAC TTT CTT GTT CAG AGT CTG AAC GTC AGA	Mutate alanine at site 18 in rLpp to lysine
lppA18K Rev	TCT GAC GTT CAG ACT CTG AAC AAG AAA GTT GAC CAG CTG AGC AAC	
LppL16K For	CTG GTC AAC TTT AGC GTT CTT AGT CTG AAC GTC AGA AGA C	Mutate leucine at site 16 in rLPP to lysine
LppL16K Rev	GTC TTC TGA CGT TCA GAC TAA GAA CGC TAA AGT TGA CCA G	

To create N-terminal truncations, overlap deletion PCR was performed using the Expand High-fidelity PCR system (Roche; Indianapolis, IN, USA). The DNA was amplified with the following protocol: 94°C-3 min; (94°C-30 s, 50°C-30 s, 67°C-6 min) for 10 cycles; (94°C-30 s, 50°C-30 s, 67°C-6 min + 15 s/cycle) for 25 cycles; and a final extension of 67°C for 7 min. Subsequent steps were carried out similarly to the SDM protocol.

### Antibody Production

Antibodies were produced in house using Balb/C mice (Harlan, Madison, WI, USA). To stimulate antibody production, 4–6 week-old animals were subcutaneously injected biweekly with a 1:1 solution of His-tagged rLpp in PBS: alhydrogel (Invivogen; San Diego, CA, USA). A total of four biweekly booster injections were performed on each mouse. Serum was collected before and after the experiment to analyze differences in reactivity against rLpp. Mice were euthanized via CO_2_ inhalation followed by cervical dislocation, and whole blood was collected in heparin-coated tubes. To separate the cellular and serum fractions, the sample was spun at 8000 times g for 10 min. Serum was saved for use in immunochemical analyses and the cellular fraction of the blood was discarded. The UND Institutional Animal Care and Use Committee approved the protocol for immunization (protocol #1406-2C). All efforts were made to minimize animal suffering.

### Enzyme-linked Immunosorbent Assays (ELISAs)

Enzyme-linked Immunosorbent Assays used to analyze binding were performed as follows. Nunc maxisorp 96-well plates (Fisher) were treated overnight at 4°C with 100 μL of 10 μg/mL Plg or plasmin in ELISA coating buffer (0.32 g Na_2_CO_3_, 0.586 g NaHCO_3_ per 200 mL, pH = 9.6). The wells were subsequently washed three times with PBS supplemented with 0.05% Tween (PBST), and the plates were blocked for at least 1 h with 300 μL of a 1% gelatin solution in PBS. The blocked plates were washed again as above before the detailed amounts of the rLpp and derived mutant proteins were added to the appropriate wells and allowed to bind for 1 h at 37°C. The plates were washed again as above and treated with αLpp mouse serum—generated as described above—at a dilution of 1:500 in PBS for 1 h at room temperature. Another wash was performed, and a secondary αMouse IgG conjugated to horseradish peroxidase (GE Healthcare; Pittsburgh, PA, USA) was added at a dilution of 1:5000 in PBS for another hour at room temperature. A final wash was performed prior to addition of Turbo TMB ELISA substrate (Thermo). Upon development of a blue color, 2N H_2_SO_4_ was added to the wells to stop activity and the plates were read at a wavelength of 450 nm on a Biotek Epoch plate reader (Winooski, VT, USA) to assess binding. All volumes used were 100 μL except for the blocking step. *K*_D_ was determined as the concentration of substrate at half of maximal binding (Floden et al., 2011).

### Western Blot

To assess antibody reactivity against our various Lpp mutants, 100 ng of each protein were run on a 12.5 or 20% SDS-PAGE gel. After migration in a gel, the bands were transferred to a nitrocellulose membrane that was subsequently blocked with 5% non-fat dried milk in TBS-Tween overnight at 4°C. After blocking, the membranes were washed three times in TBST. Mouse serum diluted 1:1000 in TBST was used to probe each protein for binding for 1 h at room temperature. This was followed by another wash and the addition of a solution of 1:12,000 diluted anti-mouse IgG-HRP. Proteins were detected using SuperSignal West Pico Chemiluminescent Substrate (Life Technologies). Images were captured with the Odyssey Fc imaging system and accompanying software (LI-COR; Lincoln, NE, USA).

### Plasminogen Activation Assay

Ninety-six-well plates were coated with 100 μl of 10μg/ml BSA, Lpp, and enolase as a positive control (Floden et al., 2011) overnight at 4°C. The plates were brought to room temperature and washed once with PBST, then blocked with 300 μL PBS + 2% BSA for 2 h at room temperature. After another three washes with PBST, 2 μg of Plg were added to pertinent wells. The plate was washed three times with PBST and human uPA activator at a concentration of 4 ng/well was added. A substrate was made by adding D-valyl-leucyl-lysine-p-nitroanilide dihydrochloride (Sigma–Aldrich; St. Louis, MO, USA) at a concentration of 0.3 mM in a solution of 64 mM Tris HCl, 350 mM NaCl, 0.15% Triton X-100, pH 7.5. The substrate was then added and incubated overnight at 37°C. The spectrophotometer employed for the ELISAs was used to detect the color change at 405 nm. All volumes used besides the blocking step were 100 μL.

### Whole Cell Binding Assay

A whole-cell binding assay was adapted from a previously published protocol (Probert and Johnson, 1998). Briefly, Plg (10 μg/mL) was immobilized onto clean glass microscope slides in PBS overnight at 4°C. Slides were subsequently washed three times in PBS for 5 min each, blocked with a solution of 3% BSA in PBS for 2 h at room temperature, then washed again. *E. coli* MG1655 (Guyer et al., 1981) was incubated with either αLpp antibodies or mouse pre-immune serum (as a control) for 30 min at room temperature, added to the slides at a concentration of 5 × 10^6^ bacteria per mL, and allowed to bind for 1.5 h at 37°C. After binding, slides were washed extensively with PBS, and bacteria were enumerated at 400× total magnification on a BX53 microscope with a darkfield filter (Olympus; Center Valley, PA, USA). The average of 10 fields of view was used to determine numbers, with multiple slides used per replicate. Numbers reported are those averages, minus the number of bacteria that were bound to untreated slides (BSA treated only).

### Statistical Analysis

Statistical analysis was performed via one-way ANOVA with a Tukey’s *post hoc* test, when applicable^2^. An asterisk on a figure represents a *p*-value less than 0.05 when compared to the appropriate control, as detailed in the figure legends. All experiments were performed at least three times with similar results between replicates. All error bars indicate SEM of the replicates used.

## Results

### Lpp Binds Plg and Plasmin in a Dose-dependent Manner

**FIGURE 1:**
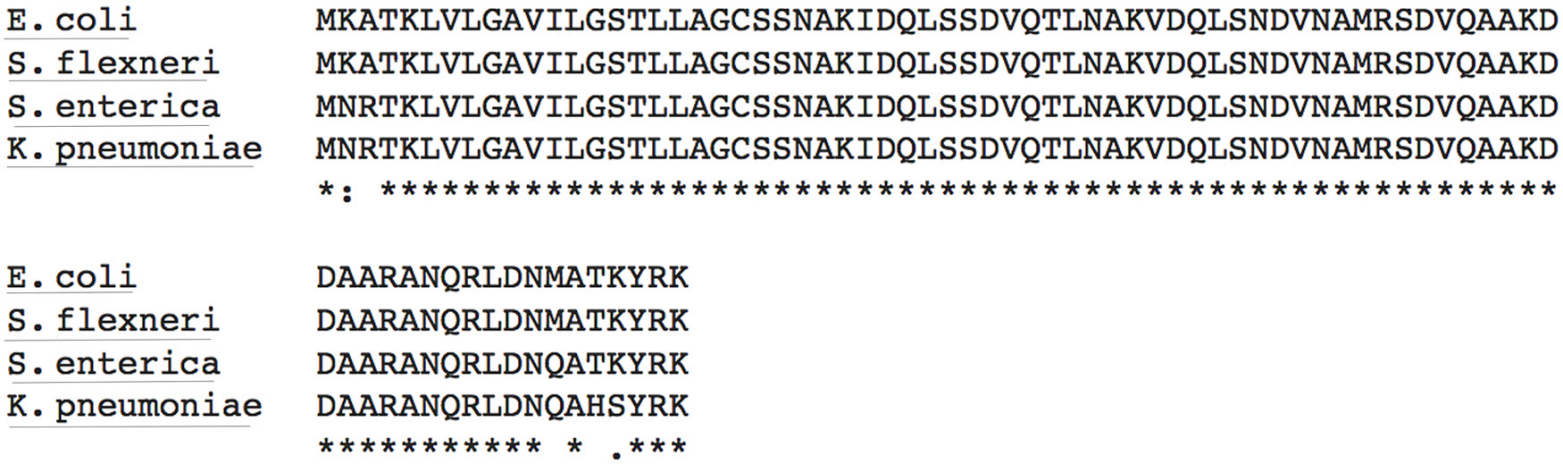
**The primary structure of lipoprotein (Lpp) in Enterobacteriaceae may allow for plasminogen (Plg) binding**. An alignment of *Escherichia coli* Lpp amino acid sequence with that of other Enterobacteriaceae using clustalW2. Accession numbers used are listed in the “Materials and Methods” section. Residues are annotated as follows: : = conservation of strong groups, • = conservation of weak groups, and * = fully conserved residue.

To begin our characterization of Lpp, we examined the primary structure for motifs that would be common in a Plg-binding protein. Many Plg receptors function via lysine-dependent mechanisms (Wiman et al., 1979; Miles et al., 1991), which led us to hypothesize that the C-terminal lysines of Lpp (**Figure 1**) could allow for the binding of Plg. Indeed, these residues reside on the outer surface of the bacterium in the current model of free-form Lpp (Cowles et al., 2011). High sequence identity was observed between Lpp and lipoproteins from various gram-negative pathogens, including this C-terminal lysine residue (**Figure 1**).

**FIGURE 2:**
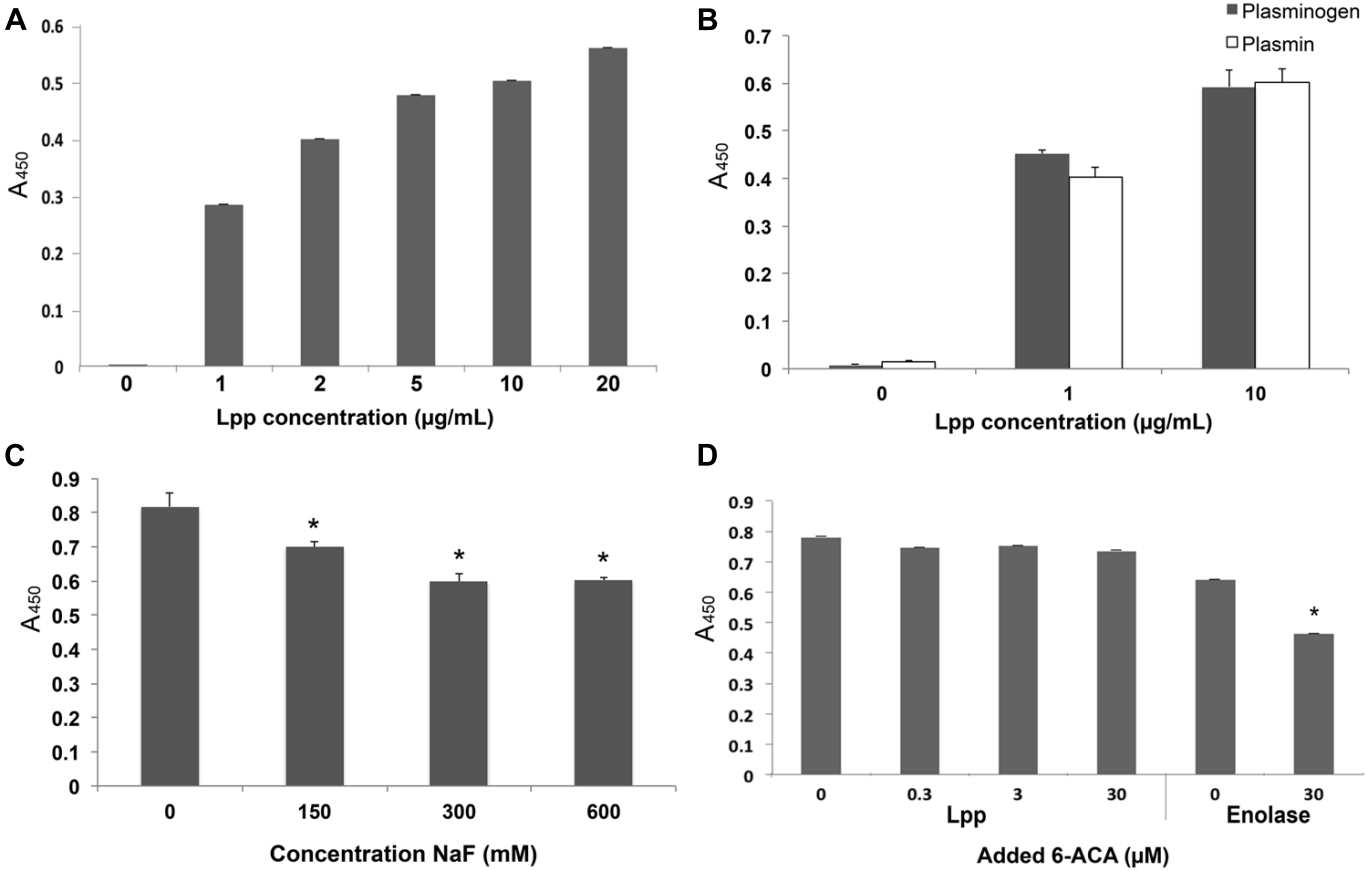
**Lpp binds Plg**. Enzyme-linked immunosorbent assays (ELISAs) were performed for experiments in which Lpp was allowed to interact with bound Plg **(A)** or plasmin **(B)** and values are blanked against those of Lpp interactions with bound BSA. The inhibitors NaF **(C)** and 6-aminocaproic acid, (ACA) a lysine analog **(D)** were used to determine the roles of ionic interactions and lysine residues, respectively. ^∗^*p* < 0.05 compared to untreated (no inhibitor) control.

Recombinant Lpp was produced minus the 21 N-terminal residues to facilitate recombinant protein production (henceforth referred to as rLpp—see **Table 1**). rLpp was examined for the ability to bind human Plg *in vitro* (**Figure 2A**). rLpp bound Plg in a dose-dependent manner, and the affinity of rLpp for Plg was relatively strong, with a calculated dissociation constant (*K*_D_) of 77 ± 16 nM, which is similar to other Plg binding proteins that we have investigated (Floden et al., 2011). Additionally, we examined the ability of rLpp to interact with plasmin (**Figure 2B**). rLpp bound plasmin, suggesting that converted Plg can still be bound by Lpp *in situ*. These results, coupled with the high level of sequence similarity between the Lpp of *E. coli* and other Gram-negative bacteria demonstrate a potential role for Lpp and homologs in *E. coli* and other bacteria in interactions with the hosts’ fibrinolytic system.

### Lpp–Plg Interactions are Inhibited at High Ionic Concentrations

We next tested the effect of various inhibitors on rLpp–Plg interactions. The ionic compound NaF was used to test the importance of ionic residues in binding reactions (Jensen et al., 2011). In principle, this reagent should interfere with ionic interactions by competitively binding acidic or basic residues. At 150 mM excess NaF, Lpp–Plg binding was inhibited by a statistically significant degree (**Figure 2C**). This result suggests that ionic interactions may be important in Lpp–Plg interactions. We then tested the ability of the lysine analog aminocaproic acid (6-ACA) to interfere with Lpp–Plg binding. No reduction in binding between Lpp and Plg occurs after addition of relatively high levels of 6-ACA (**Figure 2D**). In contrast, 6-ACA decreased binding of a control Plg-binding protein from *Borrelia burgdorferi* (enolase; **Figure 2D**.). These results suggest that ionic interactions play a role in Lpp–Plg binding. However, contrary to our initial hypothesis, the data obtained suggest that the C-terminal lysine of Lpp may not be involved.

### Truncations of the Lpp C-terminus do not Affect Lpp–Plg Binding

**FIGURE 3:**
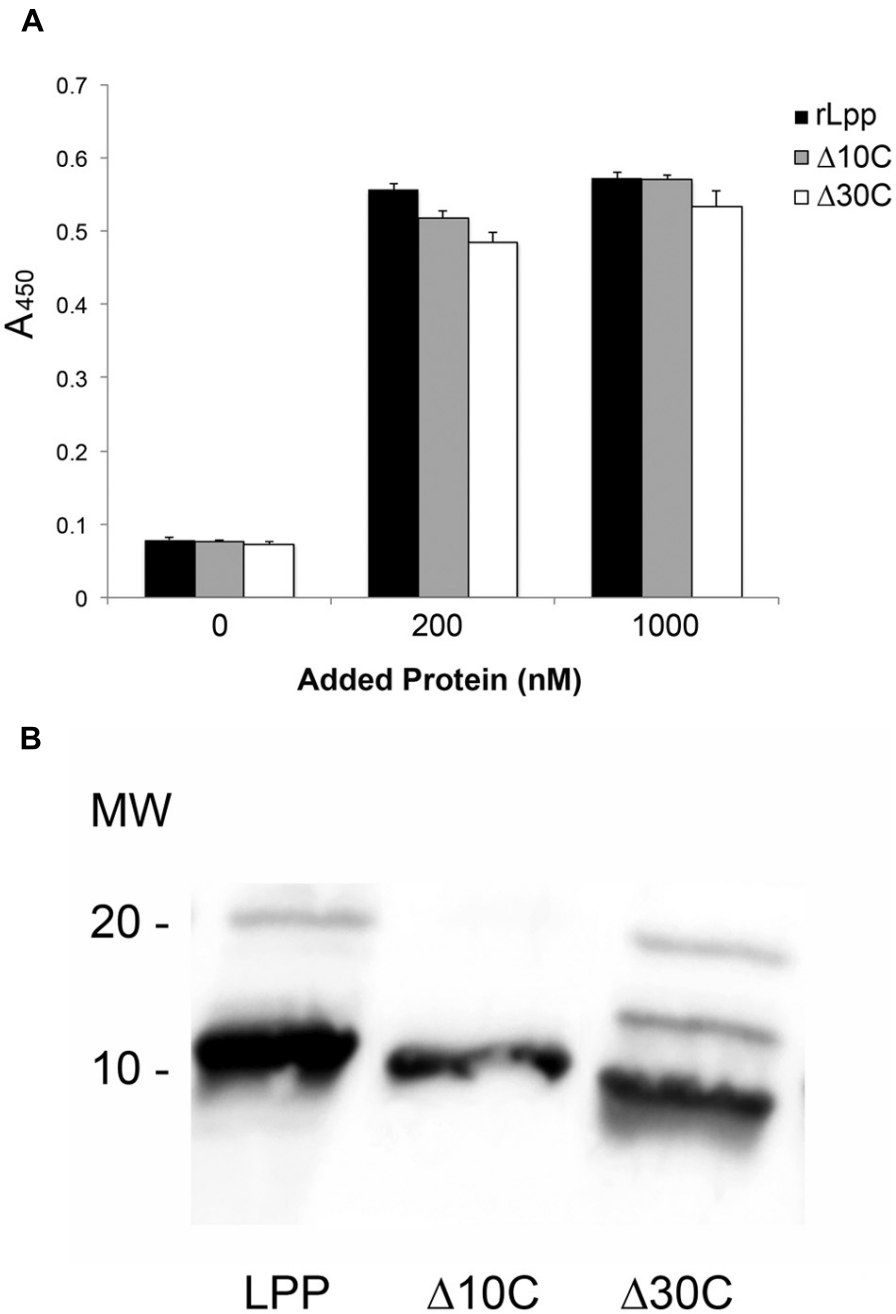
**Lpp binds to Plg independently of the Lpp C-terminal domain**. ELISA was performed to analyze the binding between Plg and Lpp C-terminal truncations **(A)**, and a Western blot was performed to verify that the mutants used retained receptivity to the αLpp antibodies generated during this study **(B)**. No statistically significant differences were observed between Lpp and C-terminal truncation mutants’ binding to human Plg.

According to the current model for free-form Lpp localization, only the extreme C-terminus is available to the extracellular environment of *E. coli*, and, therefore, is the most likely site to facilitate interaction with ECM components like Plg. Although a lysine analog did not affect rLpp–Plg binding, ionic residues may play a role (**Figure 2C**). We performed SDM to produce an rLpp mutant with a non-sense mutation 10 residues upstream of the C-terminus (**Table 2**). We assayed the ability of this mutant to bind Plg. Again, contrary to our hypothesis, rLppΔ10C was able to bind Plg at an affinity equivalent to full-length rLpp, suggesting that the actual Plg-binding domain of Lpp is located elsewhere in the lipoprotein (**Figure 3A**). To verify that the Lpp mutants could still interact with the anti-rLPP antibody, we performed a Western blot against the truncated peptide. As shown in **Figure 3B**, the truncated peptides demonstrated comparable reactivity to antibodies generated against the full-length rLpp (**Figure 3B**).

**Table 2:** A list of recombinant peptides generated in this study and their affinities for human plasminogen (Plg).

Peptide name	Sequence	Change in binding vs. normal
rLpp	SSNAKIDQLSSDVQT**LNAKVDQLSNDV**NAMRSDVQAAKDDAARANQRLDNAATKYRK	N/A
rLppΔ10C	SSNAKIDQLSSDVQTLNAKVDQLSNDVNAMRSDVQAAKDDAARANQR	None
rLppΔ30C	SSNAKIDQLSSDVQTLNAKVDQLSNDV	None
rLppΔ10N	SDVQTLNAKVDQLSNDVNAMRSDVQAAKDDAARANQRLDNAATKYRK	None
rLppΔ20N	DQLSNDVNAMRSDVQAAKDDAARANQRLDNAATKYRK	Decrease
rLppΔ30N	RSDVQAAKDDAARANQRLDNAATKYRK	Decrease
rLppK19A	SSNAKIDQLSSDVQTLNAAVDQLSNDVNAMRSDVQAAKDDAARANQRLDNAATKYRK	Increase
rLppQ14A N17A	SSNAKIDQLSSDVATLAAKVDQLSNDVNAMRSDVQAAKDDAARANQRLDNAATKYRK	Increase
rLppL16R A18K	SSNAKIDQLSSDVQTRNKKVDQLSNDVNAMRSDVQAAKDDAARANQRLDNAATKYRK	Decrease
rLppA18K	SSNAKIDQLSSDVQTLNKKVDQLSNDVNAMRSDVQAAKDDAARANQRLDNAATKYRK	None
rLppL16K	SSNAKIDQLSSDVQTKNAKVDQLSNDVNAMRSDVQAAKDDAARANQRLDNAATKYRK	None

*Bolded text indicates putative Plg-binding domain*.

### The Plg-binding Domain is Near the Center of the Lpp Molecule

**FIGURE 4:**
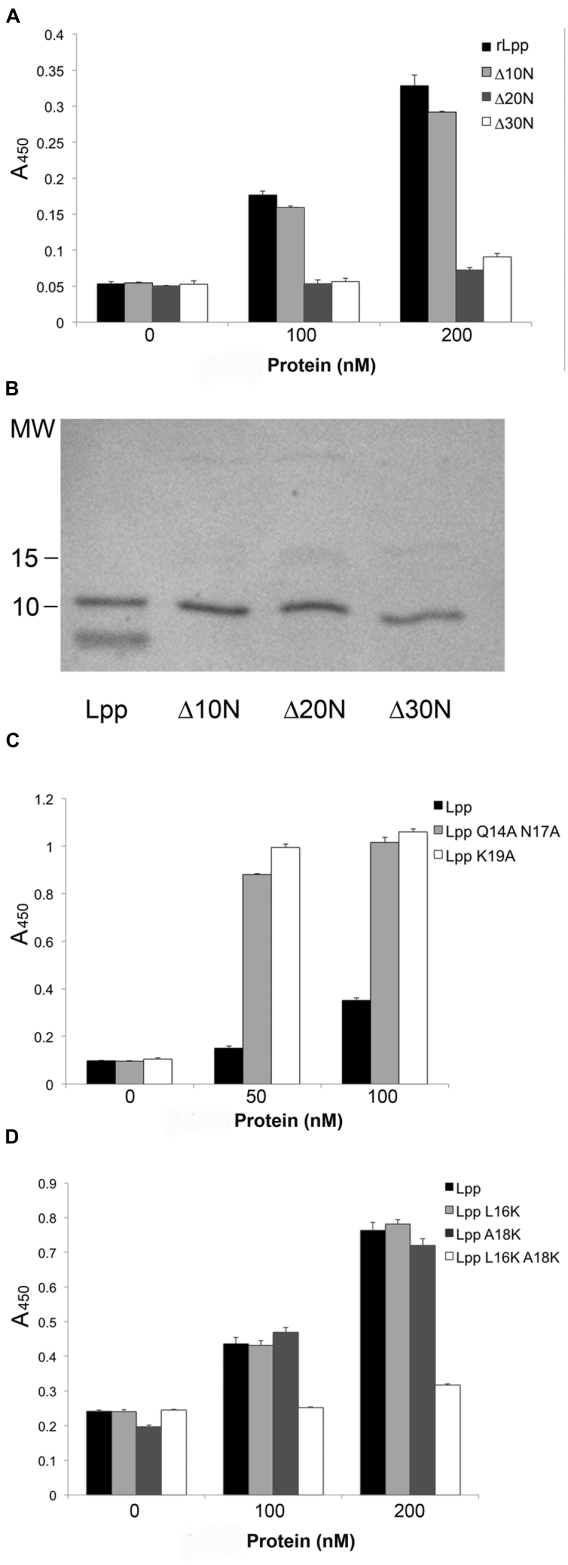
**Neutral residues near the N-terminus of Lpp are required for optimal binding of Plg by Lpp**. The N-terminal truncations of Lpp were analyzed via ELISA for binding to human Plg **(A)**, and their ability to be recognized by our αLpp was confirmed by Western blot **(B)**. ELISA was performed to assess binding of Lpp by site-directed mutagenesis variants lacking ionic **(C)** as well as non-polar amino acids **(D)**.

The discovery that Lpp–Plg binding occurs independent of the extreme C-terminus prompted the generation of additional Lpp truncations. Firstly, a 30-residue C-terminal truncation was created, and was also found to bind Plg equivalent to the parent rLpp (**Figure 3A**). To generate truncations of the N-terminus while leaving the C-terminal domain intact, we utilized overlap deletion PCR (Lee et al., 2010) to remove 10, 20, and 30 residues from the N-terminus of rLpp. As the rLpp is a small protein, between the N- and C-terminal truncations generated, every potential binding region of the protein was analyzed. We observed a distinct drop in affinity between the 10 and 20-residue truncations (**Figure 4A**). To verify that the Lpp mutant interacted with the generated antibodies, we performed a Western blot against the truncated peptides, and noted no lack or increase of binding when compared to full-length rLpp (**Figure 4B**). These data suggest that at least part of the Plg-binding site of Lpp is located between residues 10 and 20, and ends before V27, as C-terminal truncations up to that reside did not negatively impact binding.

As we were able to reduce binding earlier with ionic additives (**Figures 2C,D**), we hypothesized that charged residues might play a role in the interaction. To test this hypothesis we performed SDM on several residues in the residue 10–20 domain. The mutation of three residues in particular—Q14, N17, and K19—actually caused an increase in the affinity of Lpp for Plg (**Figure 4C**). With this new information taken into account, we decided to target the hydrophobic residues in this region. We were able completely eliminate detectable binding by mutating the L16 and A18 residues to either acidic or basic amino acids. However, when the residues were mutated individually, no phenotype was seen, suggesting a compensatory role in Plg binding (**Table 2**). Altogether, the data suggest an importance for hydrophobic residues in the interaction of Lpp with Plg.

### Plg Bound by Lpp can be Converted to Active Plasmin

**FIGURE 5:**
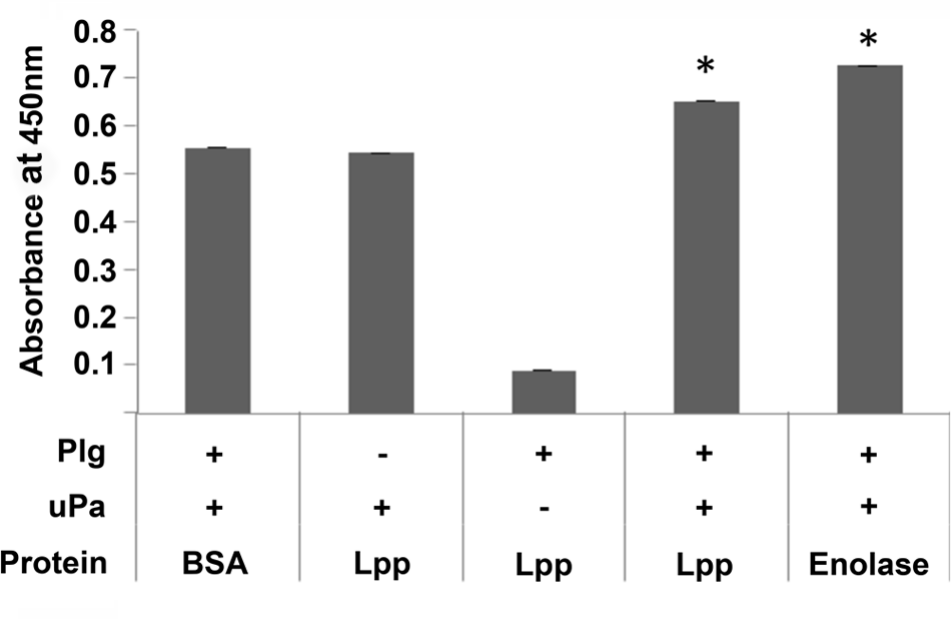
**Plg bound by Lpp can be converted to active plasmin by uPA**. A Plg activation assay was performed using BSA (negative control), Lpp, and borrelial enolase (positive control) as coating proteins and human urokinase plasminogen activator (uPA) as the activator. ^∗^*p* < 0.05 when compared to BSA negative control value.

An interaction with a binding protein may result in a conformational alteration of Plg (Fulde et al., 2013). This conformational change allows Plg to be recognized and activated to plasmin by host factors tPA and uPA (Lähteenmäki et al., 2005; Lin and Hu, 2014), resulting in a functional protease. To test whether Lpp-bound Plg could be converted to active plasmin, a chromogenic assay (Floden et al., 2011) was employed in the presence of Lpp-bound Plg and uPA. Our results indicate that, when bound by Lpp, Plg could be converted to active plasmin (**Figure 5**). Altogether, these data suggest that an alternative role for Lpp may be as a functional Plg receptor on the surface of *E. coli*.

### Anti-Lpp Antibodies do not Interfere with Whole *E. coli* Binding to Immobilized Plg

**FIGURE 6:**
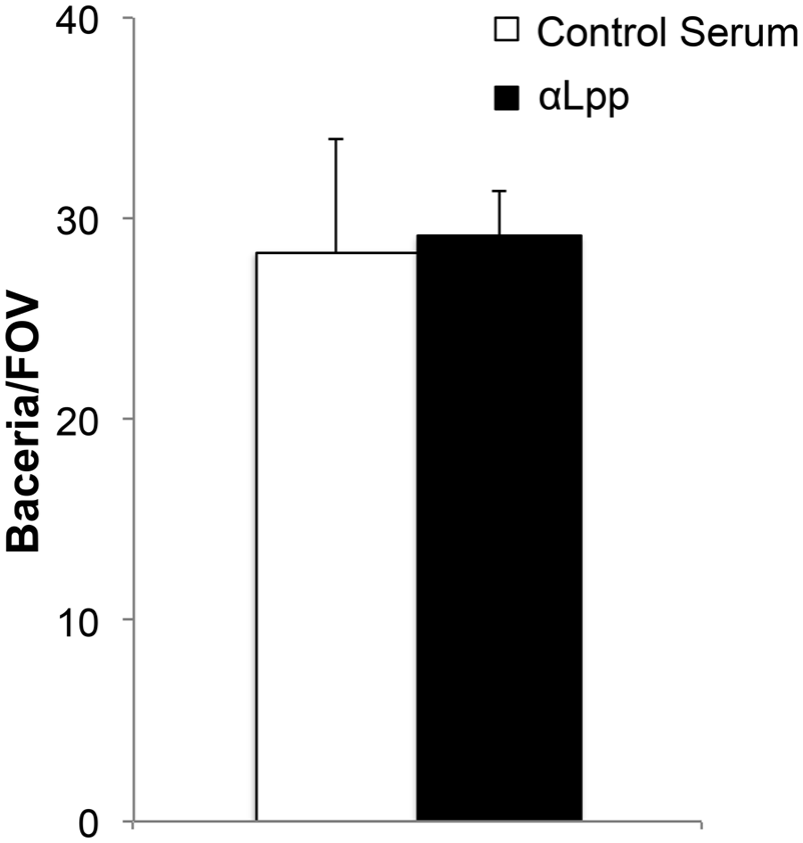
**Antibodies against Lpp do not interfere Plg with binding to whole cells**. *E. coli* were treated with αLpp antibodies or pre-immunized mouse serum (control). Binding was quantitated by bacteria/field of view (FOV) by dark field microscopy. No difference was seen comparing the two groups with regards to Plg binding.

To examine the functionality of Lpp–Plg interactions in intact *E. coli*, we performed a binding assay using dark field microscopy. No difference was seen with *E. coli* binding to immobilized Plg on glass slides even after treatment with αLpp antibodies up to a dilution of 1:50 in the bacterial suspension (**Figure 6**).

## Discussion

A location for the free form of *E. coli* Lpp was recently proposed (Cowles et al., 2011), and although a definitive function for this protein form has not been delineated, several potential roles have been postulated. Whatever the true function of free-form Lpp, it is likely to be conserved between at least several species of Enterobacteriaceae due to the highly conserved nature of the protein (**Figure 1**). Lpp has a critical role in infection likely because bacterial mutants lacking in all forms of Lpp are severely structurally compromised even under ideal culture conditions (Sha et al., 2004). By studying the specific domains of the protein, we may be better able to specifically understand the importance of free-form Lpp in bacterial pathogenesis.

In our investigation of the protein–protein interactions, we were surprised by the nature of the described binding activity. Most proteins that interact with Plg are highly affected by the addition of ionic inhibitors such as sodium salts and heparin, as the interactions are mostly mediated through charged amino acids (Walker et al., 2005; Fulde et al., 2013). However, ionic compounds were required in relatively high concentrations to have the anticipated effect (**Figure 2C**). Moreover, many proteins that can bind Plg depend on C-terminal lysine residues to facilitate the interaction with Plg (Miles et al., 1991; Brissette et al., 2009; Floden et al., 2011; Bhattacharya et al., 2012; Önder et al., 2012). Removing up to 30 residues from the C-terminus of Lpp had no effect on the protein’s ability to bind Plg (**Figure 3**). Studies have shown that other bacterial proteins have non-terminal Plg binding domains that may use other amino acid motifs, most notably positively charged residues in a hydrophobic pocket (Walker et al., 2005; Sanderson-Smith et al., 2007). Lpp naturally forms multimers in intact bacteria (Shu et al., 2000) which are clearly visible in **Figure 3B**. The possibility remains that in our purification of rLpp and the various mutants from *E. coli* that wild-type Lpp was also purified, thereby confounding our results. However, the mobility of full-length Lpp and the truncation mutants (**Figure 3B**) argues against significant wild-type LPP contamination in our protein preparation. The results of our assay utilizing 6-ACA (**Figure 2D**) bolster our confidence in the conclusion that the extreme carboxyl terminus of Lpp (i.e., the C-terminal lysines) is not involved in Lpp–Plg interactions. Future investigations may be necessary to verify these findings.

By scanning from the N-terminus of our rLpp with 10-residue truncations, we found a domain that we hypothesized could be involved in Plg binding. Mutation of the asparagine and lysine residues in this pocket, however, yielded a protein that had a higher affinity for Plg, while mutation of the hydrophobic residues resulted in an Lpp mutant completely unable to interact with this host zymogen (**Figures 4C,D**). To reconcile these data with those obtained earlier (**Figures 2C,D**), we hypothesize that the addition of ionic compounds to the binding buffer may interfere with interactions by disrupting the tertiary structure of either Lpp or Plg at the pertinent domain. Additionally, our mutation of charged residues in the examined domain may negatively affect the structure of the binding site by altering the charge of the overall binding region. As discussed above, a hydrophobic pocket is required for this alternative binding strategy of atypical Plg-binding proteins (Walker et al., 2005; Sanderson-Smith et al., 2007).

**FIGURE 7:**
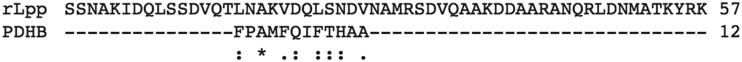
**The putative Plg-binding domain of rLpp is similar to that of another moonlighting protein**. A Clustal W2 alignment of rLpp and a previously described Plg-binding peptide from PDHB of *Mycoplasma pneumoniae*.

Of note, the putative location of the Plg binding in rLpp is localized in this study to the domain ^16^LNAKVDQLSNDV^27^ the N-terminus of this peptide was determined by the SDM in **Figure 4**, and the C-terminus was the last amino acid not affected by the large truncations in **Figure 3** (bolded on the Lpp sequence in **Table 2**). This sequence is similar to a previously identified Plg-binding domain identified on the pyruvate dehydrogenase subunit B from *Mycoplasma pneumoniae*: ^91^FPAMFQIFTHAA^102^ (Thomas et al., 2013). In fact an alignment of the *M. pneumoniae* peptide with full-length rLpp yields an overlap at exactly that domain (**Figure 7**). While only one residue was identical between the two sequences, most other residues were annotated as conserved in function. This result is particularly interesting, as PDHB is another “moonlighting” protein found in both the inner and extracellular compartments of the bacterium (Henderson and Martin, 2011). Under the model for free-form Lpp localization that has been recently described, the proposed Plg-binding domain would be unavailable for interactions with host molecules, as only the extreme C-terminus of the protein is proposed to be exposed (Bernstein, 2011; Cowles et al., 2011) when free-form Lpp is bound to bacterial outer membrane. The model for subcellular localization of Lpp was based on the biotin labeling of outer-membrane proteins in *E. coli*. With the deletion of the Lpp C-terminus, the authors saw a complete elimination in the biotinylation of the protein. However, examination of the structure of Lpp reveals an extremely well-organized α-helix structure with a disorganized C-terminal end (Shu et al., 2000). The biotinylation reagent used in this study may only have been able to effectively label those disorganized ends, which would explain the specificity of labeling without the necessity that the remainder of the protein be sequestered within the bacterial outer membrane. In that study, the authors also addressed the issue that trimeric Lpp would not be able to insert into a membrane (Shu et al., 2000; Cowles et al., 2011). Further studies will be needed to elucidate the exact positioning of Lpp in the outer membrane, though the evidence to date strongly supports the current model.

Additionally, our experiments indicated no difference in Plg binding for intact *E. coli* when cells were treated with αLpp antibodies generated in our lab. This could be due to a number of factors. Firstly, *E. coli* has additional proteins that facilitate Plg binding (Parkkinen and Korhonen, 1989; Sjöbring et al., 1994). The role for multiple, independent Plg binding proteins has yet to be determined, but could allow for diverse functions of the different proteins, or simply allow for some proteins to be lost, in a case of classic redundancy. In fact, our results indicate that the other Plg-binding proteins may be able to bind this host protein simultaneously with Lpp, as the normal inhibitor 6-ACA had no effect on Lpp–Plg interactions. Simultaneous binding of a single host protein by multiple bacterial effectors could have a number of potential roles/functions, including potentially allowing for a tighter binding *in vivo* than can be observed *in vitro*.

Another explanation for the result presented in **Figure 6** is that Lpp is not exposed on the surface of intact *E. coli*; thereby further supporting the current model of Lpp localization. If free-form Lpp is, in fact, integrated into the membrane and not available in intact bacteria, there still exists a possible role for the protein. The outer membrane of some bacteria can be highly volatile, with a rapid turnaround rate for molecules embedded therein (Manning and Kuehn, 2013; Kulkarni and Jagannadham, 2014). For example, it is well known that LPS can be found floating freely in infected tissues. It is conceivable, then, that free-form Lpp may be released from the membrane where it could act as a Plg-binding protein. While this phenomenon would preclude Lpp from recruiting the Plg to the bacterial cell surface in the absence of another receptor, Lpp may still play a role in plasmin activation in the environment surrounding the invading *E. coli*. Indeed, Lpp binding still allows for activation of Plg to plasmin by uPA (**Figure 5**) and may actively bind that converted plasmin (**Figure 2C**). Outer membrane vesicles could clear a path for *E. coli* and other Enterobacteriaceae by acting as decoys for host immune responses and by facilitating dissemination via Lpp–Plg degradation of extracellular matrix (Ellis and Kuehn, 2010).

Although we found that Lpp can bind Plg, many aspects of the interaction still remain unknown. While we feel confident that the interaction does not depend on the C-terminal residues, elucidation of the structure from the newly created mutants may help us further identify the residues important for interactions. *E. coli* Lpp mutants already exist that are unable to produce either the free or bound forms of Lpp (Zhang and Wu, 1992; Cowles et al., 2011). With these new mutants we may be able to further differentiate between the functions of the two Lpp subtypes without having to completely eliminate either species from the cell. In fact, a more targeted approach could employ the mutants created during this study (**Figure 4**) to further identify the role of the described Plg binding. Finally, we also hope to further add to the knowledge of Lpp localization in the cell, possibly via the use of monoclonal antibodies to specific Lpp domains.

## Conflict of Interest Statement

The authors declare that the research was conducted in the absence of any commercial or financial relationships that could be construed as a potential conflict of interest.

## Footnotes

1 www.genomics.agilent.com/primerDesignProgram.jsp

2 http://vassarstats.net/anova1u.html
